# FIR agreement Indonesia – Singapore: What are the legal implications?

**DOI:** 10.1016/j.heliyon.2024.e29708

**Published:** 2024-04-15

**Authors:** Adhy Riadhy Arafah, Aktieva Tri Tjitrawati, Alifia Nuril Bais, Firnida Hanan Nurkhalisha

**Affiliations:** Faculty of Law, Universitas Airlangga, Indonesia

**Keywords:** Air navigation services, Delegation, Responsibility

## Abstract

This paper explores the implications of Indonesia's agreement to delegate the Flight Information Region (FIR) of air navigation services to Singapore, specifically in the airspace of the Riau and Natuna islands, as stated in Presidential Regulation Number 109 of 2022. Using the normative juridical method, this study examines the intricate details of the agreement, with a focus on its potential legal consequences for Indonesia's national and international obligations.

Although Indonesia retains sovereignty over its territory, this analysis scrutinizes the nuanced provisions of the agreement and their implications, particularly in terms of the technical aspects of air traffic services provided by the Singapore Air Traffic Services Provider above Indonesia's territories, which the agreement does not explicitly delineate the responsibilities or shared consequences in law. The purpose of this paper is to elucidate Indonesia's increased responsibility under the new agreement and emphasize the importance of a careful approach to its implementation. By exploring the multifaceted dimensions of national and international interests, this study seeks to highlight the imperative for Indonesia to navigate the agreement prudently.

Ultimately, this analysis aims to provide a comprehensive understanding of Indonesia's obligations, potential challenges, and essential considerations under the renewed agreement of 2022, underscoring the significance of a balanced approach in safeguarding Indonesia's interests on both national and international fronts.

## 1 Introduction

With a large territory and strategic position in the Southeast Asia region, Indonesia significantly contributes to aviation safety activities. However, the fulfillment of the safety standards in air navigation services within its jurisdiction can be challenging. In regard to its air navigation services, which entail international safety standards and jurisdiction over its own air space, Indonesia decided for the first time in the International Civil Aviation Organization (ICAO) meeting to delegate its 'jurisdiction' in air navigation services over the Natuna and Riau islands to Singapore in 1973. This decision arose from the 1st Regional Air Navigation (RAN) Meeting for the Asia-Pacific Region in Honolulu [1].

Over the course of more than three decades after that delegation, Indonesia has made several attempts to reclaim it in several RAN Meetings but has been unsuccessful. The recent enactment of the Indonesian Aviation Act (Act Number 1 of 2009) leaves the government no choice but to reclaim national control, mandating that the Indonesian National Air Traffic Service Provider (LPPNPI) assume responsibility for the services delegated to Singapore by 2024 as stated in Article 458:

“The airspace of Indonesia, where had been delegated to other countries under the agreement, must be evaluated and served by the National Navigation Service Provider no later than fifteen years after this Law applies.”

The first delegation in air traffic services above the Natuna and Riau islands was legally formalized through the 'Agreement Between the Government of the Republic of Singapore on the Realignment of the Boundary Between the Singapore Flight Information Region and the Jakarta Flight Information Region' in 1995 and it was successfully ratified into Indonesian national Law in Indonesian President Decree Number 7 of 1996. This agreement, in adherence to international requirements as a source of international law, carries a fulfillment according to the Statute of the International Court of Justice, Article 38 (1), which states.

1. International conventions, whether general or particular, establishing rules expressly recognized by the contesting states;
2. International custom, as evidence of general practice accepted as law;
3. The general principles of law recognized by civilized nations;
4. Subject to the provision of Article 59 of the statute, judicial decisions, and the teachings of the most highly qualified publicists of the various nations as subsidiary means for the determination of rules of law.”

Furthermore, the most practiced sources of international air law are treaties, conventions, and other instruments that are binding under international law, multilaterally and bilaterally. Therefore, the parties of the agreements have legally binding obligations in international law to which the treaty creates the rights and responsibilities of the parties in the agreement ([2]:8). In the case of the Überlingen accident, the German government and Swiss air navigation provider company, Sky-Guide, made a joint decision to manage the airspace above Überlingen. However, they had not yet signed the Letter of Agreement that typically aligns with the principles established by the International Treaties Convention of 1969 (VCLT). Unfortunately, this decision had legal ramifications when the accident occurred [3].

Nevertheless, while this historical context and legal framework are established, this study aims to delve deeper into the implications for Indonesia under international law arising from the ratified agreement between Indonesia and Singapore in 2022. To bolster the foundation of this inquiry, this paper will address a complete description of relevant cases, a comprehensive literature review within the domain, and a focused elucidation of objectives and limitations. It will scrutinize the legal consequences and obligations concerning the agreement, aiming to offer a meticulous analysis rooted in both historical context and international legal principles.

## 2 Methodology

This research aims to explore the consequences of the agreement between Indonesia and Singapore, which was ratified in 2022, in light of the established historical and legal framework within the context of international law. To deal with this matter in a comprehensive manner, the study adopts a normative juridical methodological approach.

This study employs a conceptual approach and primarily uses the normative juridical method to examine the international legal framework established in the Chicago Convention of 1944 on International Civil Aviation. The analysis also evaluates the implications of the delegation agreement in navigation services in 2022 between Indonesia and Singapore on the issue of sovereignty within the airspace of the respective states.

To understand the legal landscape, this conceptual framework relies on a comprehensive review of primary resources, including international conventions, national laws related to aviation, and international standards and recommended practices. Furthermore, the foundational understanding of the legal landscape is complemented by secondary sources, such as scholarly books, peer-reviewed journals, and credible online news reports.

## 3 Exploring air navigation services delegation and state sovereignty in international aviation law

The authority of a state to exert control over its territory (jurisdiction) is closely tied to its sovereignty, which is the most critical aspect of international law principles. The right, furthermore, in air law, is recognized in Article 1 of the Chicago Convention 1944 on International Civil Aviation (Chicago Convention 1944). Based on this legal principle, every state in the world shall respect the sovereign rights of other states [4].

Within the realm of jurisdiction, a state is granted the power to exert authority over all individuals and entities within its territorial limits, including the provision of air navigation services. As air space activities transcend borders, it is universally recognized that a state's jurisdiction may extend to aircraft operating within the territories of other states [5].

However, aviation operations are not limited to a single territory, but are also classified as a transnational activity. Therefore, when aircraft flights cross into another territory, they require guidance from ground officers. The state below the air space is responsible for providing the service to the aircraft, as mentioned in Article 28 of the Chicago Convention 1944:

“Each contracting State undertakes, so far as it may find practicable, to:

Provide, in its territory, airports, radio services, meteorological services and other air navigation facilities to facilitate international air navigation, in accordance with the standards and practices recommended or established from time to time, pursuant to this Convention;”

ICAO member States are obligated to standardize air navigation services at aerodromes and in air space, as per Annex 11 of the Chicago Convention 1944. This requirement necessitates the provision of air navigation services in the specified areas, which should conform to the established standards [6]. Article 25 of the Chicago Convention 1944 mandates that the principle of determination also applies to aircraft in distress. Furthermore, it stipulates that every member state of the International Civil Aviation Organization (ICAO) must provide any feasible measures of navigation assistance to distressed aircraft flying within its airspace.

There exist various reasons why numerous nations opt to transfer their responsibilities to other states or international organizations. These reasons may include geographical positioning, effectiveness, and capability to offer services. In situations where a state chooses to delegate its responsibilities of serving and providing air navigation services in the airspace, a cross-border air navigation services agreement must be established to stipulate the provisions of the delegation ([7]:95). Thus, agreements between two countries or between countries and international organizations should consider the interests of transboundary or air traffic services. This may include the option to transfer authority or legal enforcement competencies, as informed by other states. The agreements should be formal and fair, accommodating the needs of all parties involved.

The obligation to make an agreement in the delegation of air traffic services is stated in Standard 2.1 of Annex 11 (Air Traffic Services) of the Chicago Convention 1944:

“Contracting States shall determine … those portions of air space and those aerodromes where air traffic services will be provided. They shall thereafter arrange for such services to be established and provided … except that, **by mutual agreement**, a State may delegate to another State the responsibility for establishing and providing air traffic services in flight information regions, control areas or control zones extending over the territories of the former.”

It is essential to emphasize that the phrase “by mutual agreement” implies that both parties, the delegating state, and the delegated state, must derive mutual benefits from the agreement. This reciprocal benefit may manifest in various risk forms, such as economic, political, social, or strategic benefits. Moreover, the ‘notes’ of this provision does not regulate the status and condition of the sovereignty of the delegating state by stating:

*“If one state delegates to another state the responsibility for the provision of air traffic services over its territory, it does not without derogation of its national sovereignty. Similarly, the providing State’s responsibility is limited to technical and operational considerations and does not extend beyond those pertaining to the safety and expedition of aircraft using the concerned air space*."

The explanation provided in the ‘notes’ [8] indicates the potential issues of sovereignty that might be discussed or asked during negotiation or after the delegation agreement has been signed. Hence, the parties of the agreement shall consider the national interest of each party in relation to their international obligation for air safety.

In Europe, for example, there are numerous cross-border agreements in which states take part. These agreements adhere to a shared principle that stipulates that a specific airspace area can only be governed by a single air traffic service provider at any given moment. Additionally, the agreements are negotiated and ratified at a different level of government or sometimes negotiated between two air traffic service providers without government intervention. In such cases, the agreement is not qualified as an international public bilateral or multilateral agreement, instead is qualified as an international private bilateral or multilateral cross-border agreement [9].

The agreements in question were established through practical measures, and their implementation was effective without requiring the full involvement or formal recognition of governments. These agreements were primarily related to air traffic services, and the entities involved did not see the need to regulate them formally as bilateral or multilateral agreements between governments. It was acknowledged by both parties that these transboundary agreements were only loosely connected to legal circumstances and were more focused on practical cooperation and mutual benefits [10]. Despite the lack of formal recognition, the agreements were successfully implemented and played a significant role in facilitating air traffic between the relevant regions.

Though an agreement is necessary for air navigation delegation, there is currently no particular international agreement model governing the transfer of Air Traffic Services between countries or international organizations. Typically, existing agreement models follow the principles outlined in the International Treaties Convention of 1969 (VCLT). As such, signing or ratifying an agreement in accordance with international law automatically binds the parties to the agreement, as per the principle of *pacta sunt servanda* [11].

## 4 The agreement on the delegation of Indonesia air navigation services to Singapore

The agreement between Indonesia and Singapore is a fundamental constitution for delegating air traffic services. In order to extend the services of an Air Traffic Control (ATC) provider to a neighboring sovereign territory for operational reasons, a bilateral agreement must be established and approved by both involved parliaments. This is a critical matter as it pertains to the extension of a nation's sovereign power into foreign territory. Allowing the execution of foreign sovereign power on another state's territory can have significant implications for the fundamental nature of a nation ([12]:55).

The transfer of air traffic services to another country or entity is a widespread practice that aims to maintain a safety and efficient flow of air traffic across different nations. This is particularly important when dealing with high-speed air traffic that travels through the airspace of multiple countries. The delegation in these operations pertains to the provision of air traffic services in a particular area of their airspace to other providers of air traffic services. These providers have received an operating license and are subject to regulatory compliance and level enforcement capacities ([13]:35–42). Moreover, the delegating state's exercise of authority is limited to national requirements related to air traffic services.

Under Article 263 of the Indonesian Aviation Act 2009, several reasons which justify the delegation of national air traffic services to other countries are.

1. Flight route structure;
2. Air traffic flows; and
3. Aircraft movement efficiency

However, the transfer of air traffic services from Indonesia to Singapore above the Riau and Natuna islands is based on the historical precedent established during the first Asia – Pacific Regional Air Navigation (RAN) Meeting in 1973 in Honolulu, United State of America (USA). As per the resolution of the inaugural World Air Navigation Meeting in 1946, the meeting decision was to convene a pragmatic and consultative forum aimed at charting out a comprehensive roadmap of the challenges and protocols related to the authorization and technology of air traffic amenities and services that are essential for global air transport in the designated region [14].

The Regional Air Navigation (RAN) meetings for the Asia-Pacific were held every ten years from 1973 until 1993. In general, the Annual Meeting of Air Navigation is divided into several regional geographical positions such as African (AFI); Asia-Pacific (ASIA/PAC); Caribbean (CAR); European (EUR); Middle East (MID); North-American (NAM); North-Atlantic (NAT); and South-American (SAM) [15]. The meetings serve as a place to discuss the problems and developments of air safety, standards, and rules, including a forum for its members to delegate or reclaim their rights in air traffic control to or from another state. Furthermore, regional Air Navigation Plan (ANP) issues are also discussed or amended during these meetings, which are partly subject to the ICAO Council's approval. Nevertheless, the ICAO currently acknowledges the updating of an ANP by rotating the draft revision, without necessarily convening a meeting.

Since the second Asia – Pacific RAN Meeting in 1983 [16] and the third meeting in 1993, Indonesia has consistently attempted to regain control of air traffic services states in every ICAO formal and bilateral meeting. The reasons for this are based on the reality that the delegating state (Indonesia) has always been the disadvantaged party since the delegation has been working. At the next meeting, Indonesia made an attempt to regain control of air traffic services during the third RAN meeting in 1993 that held in Singapore. At the meeting, Indonesia claimed to have fulfilled the minimum requirements for the facilities and human resources required in the second meeting. However, no decision was made on this issue during the 1993 meeting, which was also the last regional air navigation meeting [17]. The meeting also did not discuss the legal issues and consequences arising from the delegation of Indonesia's air space to Singapore without an international agreement. It was discovered that there was no legal justification for the transfer of air navigation services from Indonesia as the delegating state to Singapore as the delegated state until 1995.

The first delegation of air navigation services above Riau and Natuna islands from Indonesia to Singapore was formally signed by the “Agreement Between the Government of the Republic of Singapore on the Realignment of the Boundary Between the Singapore Flight Information Region and the Jakarta Flight Information Region” happened in 1995. The agreement was successfully ratified into national law through Indonesian President Decision Number 7 of 1996. Moreover, the agreement has been replaced by a new agreement at the beginning of 2022 and ratified by Indonesia in Presidential Regulation Number 109 of 2022.

According to Article 458 of the Indonesian Aviation Act of 2009, it is required that the FIR above Riau and Natuna island be taken over prior to 2024. To achieve this, a renewal agreement of the 1995 agreement has been signed by both governments in 2022. Upon reviewing the delegation agreement provisions of both agreements, it is clear that Indonesia's position remains largely unchanged. The agreement of 1995 in Article 2 Paragraph 2, governs Indonesia's delegation of air navigation services from the surface to an unlimited height for Sector B and up to 37,000 feet for Sector A. In addition, the 2022 agreement in Article 2 Paragraph 1 and 2, indicates that Indonesia delegated navigation services from the surface to 37,000 feet to Singapore in sectors A and B. Hence, Singapore still has effective control in giving air navigation services to all aircraft flying above the Riau and Natuna islands. However, to respond to the new agreement, Indonesia has claimed the control by not delegating to the unlimited height level for air navigation control at sectors A and B. Indonesia has successfully ‘regained the control and unified the area control’ at altitude above 37,000 feet in air navigation services above the Riau and Natuna islands.

The agreement of 2022 has a structure that comprises of two distinct levels of agreements. Upon the fact, it can be observed that the agreement has been segregated into two parts. The first agreement, signed by the Ministry of Transportation of Indonesia and the Ministry of Transportation of Singapore and has been successfully ratified by Indonesia through a Presidential Regulation that has been published to the public. The primary agreement serves as the overarching agreement for the other technical agreement. Nonetheless, the second agreement, known as the Letter of Operational Coordination Agreement (LOCA), has yet to be disclosed to the general public. While the second agreement emphasizes technical aspects, the primary legal responsibilities of the state can be located in the first agreement.

Furthermore, despite hopes that the new agreement in 2022 would address the issue of responsibility delegation, it appears that Indonesia will continue to bear the full responsibility for all navigation services activities within its airspace served by Singapore. The delegation provisions outlined in Article 2 of the agreement are solely focused on technical delegation from Indonesia to Singapore, similar to the agreement of 1995. However, there is no mention of delegation of responsibility or any legal consequences that may arise from Singapore providing air navigation services above Indonesia's territory, such as liability for recovery damages.

Therefore, it's worth noting that Singapore is potentially responsible for its services in many aspects of safety elements required by ICAO. This includes the possibility of obligation for any recovery damage associated with service failures, as well as the requirement to offer compensation if necessary. In the absence of specific provisions governing responsibility delegation from Indonesia to Singapore, Indonesia retains liability for accidents caused by technical faults from the Singapore Air Navigation operator.

## 5 Indonesia’s responsibility through their air traffic controllers

State responsibility in providing air traffic services, pursuant to Article 28 of the Chicago Convention, has been interpreted and divided into two categories by Niels van Antwerpen: The first category involves the responsibility for providing air navigation services, while the second category pertains to the responsibility of providing those services in compliance with the ICAO Standard and Recommended Practices (SARP's) ([7]:113). In the case of a failure that causes damage by the state's actions, the state's responsibility is also followed by its liability for compensation to the victim. It is important to note that the word “liability” has consequences that are not arising as a breach of international law[18]. Thus, in cases of state liability, the state is subject to its own domestic laws, whereby a fixed liability system exists and the state takes direct responsibility for damages caused by the air traffic service entity in charge ([7]:203).

Typically, the state government is responsible for providing and managing air traffic services within its airspace. As a result, any liability related to these services is linked to the state that provides them. If an accident results in losses and a claim for compensation is made, the responsible party for liability would be the state [19]. Nevertheless, the liability of the state for loss due to the air traffic controller must be permanent liability under the state, despite the entities providing services being no longer government institutions but corporatized or privatized entities [20]. This is because, according to Article 28 of the Chicago Convention 1944, the responsibility in service is provided to the state, not to the private entity.

In addition, the nature of air navigation services is commonly served by entities of the governmental structure, and the server is civil servants. In cases where government employees are responsible for providing air traffic services, the state holds accountability for the actions of its employees and agents while carrying out their official duties [21]. As a result, if an air traffic controller is a government employee, any action or inaction on their part would be considered a state act.

Presently, there is no international or regional convention which regulate the liability provisions of the ATC. The liability of the ATC is always governed by national laws, which have usually offered a satisfactory framework ([22]:51–63). Indonesian air navigation services are provided by state-owned enterprises, and therefore not administered directly by a state organ, as per Indonesian Government Regulation Number 77 of 2012.

The initiative to introduce uniformity into the liability norms governing air traffic control came in the 1960s when air traffic controllers were served solely by governmental entities. Upon reviewing the liability regimes, the legal commission concluded that there exists a notable void in domestic laws regarding state liability. This spectrum ranges from complete state liability to complete impunity of state government against all claims, including those related to air traffic control services [23].

Furthermore, the recent FIR delegation agreement of 2022 outlines specific guidelines for the placement of Indonesian Air Traffic Controllers at the Singapore Air Traffic Control Centre (SATCC), as outlined in Article 3 of this agreement. Therefore, this arrangement has implications for the broader authority of the state with regards to legal matters. This move indicates the extension of state jurisdiction beyond national territories, in compliance with international regulations on navigation services as a general trend [24]. Although jurisdiction agreements are more commonly found in cross-border commercial agreements, they also play a crucial role in cross-border air navigation services. The jurisdiction agreement specifies the enforceable rights, and the court retains the discretion to determine court jurisdiction based on procedural considerations [25].

There are concerns regarding the jurisdiction of the Indonesian-Singapore agreement as it lacks provisions to regulate the placement of the air traffic controller at SATCC. This may lead to questions regarding the legality of the Indonesian Air Traffic Controller's duty, as it is performed in Singaporean jurisdiction despite overseeing Indonesian airspace ([26]:15–18). It could be argued that this duty falls under Indonesia's obligation to provide services above its territory as outlined in Article 28 of the Chicago Convention, but is being carried out in Singaporean territory.

In addition, questions may arise regarding jurisdiction in the event of an accident caused by technical reasons during the air traffic controller's work. Which law will apply for claiming compensation - the national law of the air traffic controller's citizenship or the national law of the location where they were controlling air traffic?

Air Navigation Services refer to the services provided by a state to aircraft passing through its national airspace. These services are governed by the Law, and the rights and obligations of both the service provider and users are clearly defined in an agreement. It is important to note that the provision of air navigation services is subject to international regulations and the state's jurisdictional practices [27].

For recovering loss resulting from the negligence of the employees or staff of the providing state, the state providing the service has an obligation to compensate on behalf of its agents or staff. Regarding the application of law in a claim against the state providing the service, it is subject to domestic laws and the jurisdiction courts of that state. Furthermore, the delegating state has legal recourse to seek compensation for expenses incurred due to loss or damage caused by the negligence of the providing state, its employees, or any other authorized representative. However, such claims must be pursued in the national courts and adhere to the domestic laws of the providing country [9].

In many state practices, the air traffic controller, in doing its work, has been protected by its national Law. The protection, known as immunity [28], is conferred by applying the air traffic controller's national law of citizenship rather than the jurisdiction of other states' laws. In Indonesia, the word “immunity” as a privilege of the Air Traffic Controller is not stated clearly in national regulations. The Indonesian Aviation Act in Article 271 Paragraph 1 solely states that “The Government shall be responsible for flight navigation service operation for aircraft operated within the air space being served.”

However, at the legislative level, there is no regulation or any interpretation regarding the responsibility of the Indonesian government even in the Article 271, including the provision of Indonesian Air Traffic Controller protection. Consequently, to protect Indonesian air traffic controllers, a legislation implementing Indonesia's jurisdiction over its air traffic controllers is required, in accordance with the duties stated in Article 28 of the Chicago Convention 1944.

The absence of a clause delegating state jurisdiction in the agreement between Indonesia and Singapore for Indonesian Air Traffic Controllers placed at the Singapore Air Traffic Control Centre (SATCC) demonstrates that the Indonesian ATC is subject to Singapore's jurisdiction. Accordingly, the Indonesian government may find it difficult to establish the legal basis to protect the immunity for Indonesian Air Traffic Controllers.

## 6 The ratification of the delegation agreement under the presidential agreement

Indonesian Law on International Agreement of 2000 proposed in Article 10, “regulates any international agreement that can be ratified by Law in relation to.

a. political issue, peace, defense, and security issues of the country;
b. Changes in the territory or determination of the territorial boundaries of the Republic of Indonesia;
c. Sovereignty or sovereign rights of the state;
d. Human rights and the environment;
e. The establishment of new legal rules;
f. Foreign loans and/or grants.”

While the first delegation agreement was signed in 1995, Indonesia, as stated earlier, had ratified the agreement under the Presidential Decree of the 1996. Furthermore, the second agreement of 2022 that replaced the first agreement was also ratified at the same level in the Presidential Regulation. However, the first agreement was signed and ratified before Indonesia had established a national Law concerning International Agreements.

The ratification of air traffic services delegation issue through Presidential Decree by the Indonesian government is a technical matter and not related to national interest or sovereignty [29]. Nevertheless, this issue has a direct connection to the territory of the state below, which is a sovereign state as mentioned in Article 2 of the Chicago Convention:

“For the purposes of this Convention the territory of a State shall be deemed to be the land areas and territorial waters adjacent thereto under the sovereignty, suzerainty, protection or mandate of such State.”

The recognition of state sovereignty through its air space above the land and water territory is also stated in Article 2 Paragraphs (1) and (2) of the United Nations Convention of the Law of the Sea (UNCLOS) 1982.

(1) “The sovereignty of a state extends, beyond its land territory and its internal waters and, in the case of an archipelagic State, its archipelagic waters, to a belt of sea adjacent to its coast, described as the territorial sea.
(2) This sovereignty extends to the air space over the territorial sea as well as to its bed and subsoil.”

The fact is that the air space of Indonesia is served for its air navigation by Singapore, which means that both countries need to ratify the delegation agreement for these services. Hence, the delegation agreement will take a position as a recognition of the sovereignty of each party and the sovereign rights that may be enjoyed by both parties. In addition, Article 10 of the Indonesian Law Number 24 of 2000 on International Agreement states that ratification through Law level is required for any international agreement related to Indonesia's sovereignty or sovereign rights, as outlined in point (C), which means that the delegation agreement is not a part of Article 10.

Furthermore, the Indonesian government said that delegation is limited and has been done by around 55 countries in the world [30]. Thus, ratification through a Presidential Regulation is considered sufficient, and it is not necessary for it to be ratified by Law. Hence, the Indonesian government at the legislative process through the agreement into national law had never opened to the public until ratification by Presidential Regulation was released.

The interpretation of Article 10 of Indonesian Law Number 24 Year 2000 was provided by the Indonesian Constitutional Court decision [31]. The court stated that their interpretation stands as follows:

*Article 10 of Law Number 24 Year 2000 (Statute Book of the Republic of Indonesia Year 2000 Number 185, Supplement to the Statute Book of the Republic of Indonesia Number 4012) is contrary to the Constitution of the Republic of Indonesia of 1945 and****has no legally binding****as****long as it is interpreted that only*** the *types of international agreements as mentioned in letters a to f in Article a quo require the approval of the House of Representative so that only those types of agreements whose ratification is carried out by Law.*

According to the authors' legal analysis, the Court's ruling necessitates the Law level to ratify all international agreements, encompassing the delegation of air traffic services agreement, that have consequences for the public interest, government rulings, and legal outcomes [32].

The other reason Indonesia has agreed to Article 6 of the agreement, which allows Singapore to collect navigation services charges on behalf of the Indonesian government. This agreement does not involve any territorial rights of Indonesia. Instead, the Indonesian government will assume legal responsibility for any risk related to Singapore's air navigation services above the Riau and Natuna islands. The reason behind this is that Singapore provides the necessary services in Indonesia's territory, and Indonesia benefits from the charges collected by Singapore.

In addition, the general principle for charging navigation services states that civil aviation operations should only be charged for services and functions that are provided for, directly related to, or ultimately beneficial to them. This principle also establishes an effective procedure for users to collect charges. It is possible to benefit from a territory without providing services, but the charge for navigation services should be based on the services and functions that are provided for, directly related to, or ultimately beneficial for civil aviation operations. (International Civil Aviation 2012). This is interpreted by Francois that the charges “should not be asked to meet costs which are not properly allocable to them” [33]. In a nutshell, the air navigation charges shall include the whole cost of services. The ICAO principles for air traffic services charging the cost consists of air traffic services, credit control and enforceable recovery procedures, following transparent allocation, justification, and genuine user dialogue.

It is important for Indonesia to consider the Draft Articles on the Responsibility of States for Internationally Wrongful Acts. These articles outline the responsibility of a state for any act or omission that can be attributed to it under international law and that violates the state's international responsibilities [34]. This means that if Indonesia commits an act that is considered internationally wrongful, it will be held accountable and responsible for it. Therefore, it is crucial for Indonesia to fully understand and abide by these articles in order to avoid any legal consequences.

It is possible for nations to manage air navigation services within their airspace by utilizing government personnel and corporate or private entities. Neglecting this responsibility and accountability could be interpreted as a breach of the state's international obligations ([7]:116). As a result, the state in whose territory air navigation services are being provided is responsible for ensuring that the objectives are met in its airspace. This recognition of culpability highlights the importance of enforcing international responsibility for air navigation services.

This provision extends to situations where a state, under the administration of public law, authorizes another state to exercise its rights. In the case of the provision of air traffic services across borders, the authorizing state may choose to delegate the responsibility of air traffic services within its airspace to the air traffic service authority of another state or an international organization, thereby relinquishing its domestic jurisdiction over the matter [35]. Therefore, in the delegation of air navigation services between Indonesia and Singapore, it should also be followed by a delegation of responsibility and liability.

Although there is no clear indication about who bears the responsibility and liability in this specific matter, it's worth noting that there is a hint in the explanatory note of Article 2.1.1 Annex 11 of the Chicago Convention 1944. This note states that if a state assigns its duties for providing air navigation services within its territory to another state, it does so without derogation its national sovereignty. However, the next explanatory note utilizes ambiguous language stating that “the providing state's responsibility is limited to technical and operational considerations and does not extend beyond those pertaining to the safety and expedition of aircraft using that air space.”

Initially, it may seem that the term implies the automatic transfer of state responsibilities from the delegating state to the providing state when air traffic services are transferred. However, if a near miss or mid-air collision occurs due to an error or omission by the air traffic service provider, the state that offered the service above the airspace of the delegating state has failed to meet the safety objectives for air navigation services in that airspace [36]. Therefore, rearranging state responsibility among the parties can be formulated by bilateral or multilateral agreements where the terms of the agreement are based on the consent of the parties.

When a state chooses to assign its rights to another state, it is crucial to take into account the delegation of responsibility and liability in addition to the transfer of rights. Without also delegating responsibility and liability, the delegation may only extend to technical duties and not encompass regulatory or supervisory roles. Even if, this could lead to the delegating state retaining the power to regulate and oversee the assigned entity. However, to guarantee a comprehensive delegation of rights, it is imperative to delegate both responsibility and liability along with the transfer of rights [37].

Learning from the Überlingen case, the collision between two aircraft on July 1, 2000, the Court of German in District of Konstanz stated that German country as the below state was liable for the accident under decision of German national law, regardless of the fact that the air traffic services were served by the Swiss air navigation entity, named Sky-Guide, which is located at Switzerland, a country the neighbour of German was because the delegation agreement had not signed yet by both parties [38].

Therefore, it should be mentioned in an Article of the delegation agreement that Singapore, as the providing service country, will be responsible for any losses that result from its negligence, or the negligence of its employees or any other person acting on its behalf, with regards to Indonesia's position. Additionally, in accordance with the provisions of this agreement, the delegating state has the right to make a claim against the providing state to recover damages for any costs or harm incurred as a result of loss or damage caused by the negligence of the providing state, its employees, or any other person acting on its behalf.

Based on the complexity of the consequences of the air navigation delegation agreement between Indonesia and Singapore, it is determined that Indonesia must place the agreement under legislation rather than Presidential Regulation. This is in accordance with Indonesian Law and the decision of the Indonesian Constitutional Court. Ratifying the agreement under Presidential Regulation without a specific consensus on the responsibility and liability clause would imply that Indonesia, as the below state, is entirely responsible for any consequences that may arise from Singapore Air Traffic Control Centre (SATCC)'s provision of air navigation services. Therefore, the agreement must be ratified by Law with the approval of the House Representative.

## 7 Conclusion

The agreement between Indonesia and Singapore regarding Air Navigation Services conforms to the standards set forth in Annex 11 of the Chicago Convention 1944. Nevertheless, the agreement to delegate Flight Information Region (FIR) in 2022 lacks clarity in terms of the specific roles, responsibilities, and liabilities between Indonesia and Singapore. This has resulted in Indonesia being held accountable for services provided by Singapore's Air Traffic Control agency, without a clear understanding of shared responsibilities.

The absence of jurisdictional delegation provision for Indonesian Air Traffic Controllers working at the Singapore Air Traffic Control Centre (SATCC) raises concerns about their protection under Singaporean jurisdiction. Any technical issues or errors that occur may require investigation under the *lex delicti* principle, which could bring Singapore's jurisdiction to apply.

In conclusion, the Indonesian government established the delegation agreement through a Presidential Regulation, which appears to have circumvented the mandates outlined in Article 458 of the Indonesian Aviation Act Number 1 Year 2009, Article 10 of the Indonesian Law on International Agreement Number 24 Year 2000 *juncto* Indonesian Constitutional Court Decision Number: 13/PUU-XVI/2018. Given the hierarchy of laws where Presidential Regulations hold a subordinate position to these laws, the agreement ought to be placed on the same level as the Law.

## Data availability

No data available in the manuscript. Data included in article and references.

## CRediT authorship contribution statement

**Adhy Riadhy Arafah:** Writing – review & editing, Writing – original draft, Methodology, Conceptualization. **Aktieva Tri Tjitrawati:** Methodology, Conceptualization. **Alifia Nuril Bais:** Project administration. **Firnida Hanan Nurkhalisha:** Project administration.

## Declaration of competing interest

We have no conflicts of interest to disclose.
